# Early diet in preterm infants and later cognition: 10-year follow-up of a randomized controlled trial

**DOI:** 10.1038/s41390-021-01368-y

**Published:** 2021-02-09

**Authors:** Nicholas D. Embleton, Claire L. Wood, Mark S. Pearce, Greta Brunskill, Victoria Grahame

**Affiliations:** 1 Population Health Sciences Institute, Newcastle University, Newcastle upon Tyne, UK; 2 Newcastle Neonatal Service, Newcastle Hospitals NHS Trust, Newcastle upon Tyne, UK; 3 Translational and Clinical Research Institute. Faculty of Medical Sciences, Newcastle University, Newcastle upon Tyne, UK; 4 Complex Neurodevelopmental Disorder Service (CNDS), Cumbria, Northumberland, Tyne and Wear NHS Foundation, Newcastle upon Tyne, UK

## Abstract

**Background:**

Achieving adequate nutrition in preterm infants is challenging. The post-discharge period may be critical for influencing growth and cognitive outcomes. We studied the effects of post-discharge nutrition on childhood cognition.

**Methods:**

Preterm-born children were randomized at ~36 weeks corrected age (CGA) to either preterm formula (PTF) or term formula (TF) until 6 months, or PTF until 40 weeks CGA, then TF until 6 months (crossover group). Childhood cognition was assessed using the short form Wechsler Intelligence Scale for Children III, allowing computation of full-scale intelligence quotient (FSIQ) and four-factor index scores; verbal comprehension, freedom from distractibility (FDI), perceptual organization (POI), and processing speed (PSI).

**Results:**

Ninety-two children were recruited (mean 10.1 years). FSIQ did not differ by group. PTF-fed children had 10-point higher PSI (*p* = 0.03) compared to crossover. Faster weight gain from term to 12 weeks CGA was associated with 5-point higher FSIQ (*p* = 0.02) and four-point higher POI (*p* = 0.04). Infant head growth was positively associated with FSIQ (+3.8 points, *p* = 0.04) and FDI (+6 points, *p* = 0.003).

**Conclusions:**

While there is no long-term impact of post-discharge macronutrient enrichment on childhood cognition, greater weight and head growth in specific epochs is associated with better outcomes. Further studies are needed to determine optimal early diet in preterm infants.

**Impact:**

- Achieving adequate nutrient intakes in preterm infants before and after hospital discharge is challenging.
- Nutrient intakes prior to discharge affect later cognitive and metabolic outcomes.
- Follow-up of a randomized controlled trial shows no cognitive benefit in later childhood from a more nutrient-dense formula compared to standard formula after hospital discharge.
- Growth in the first year of life is strongly correlated with childhood cognition and emphasizes the importance of nutrition in early life.

## Background

Preterm birth represents ~10% of all births worldwide and is associated with increased risks for adverse metabolic and cognitive outcomes over the life course.^1,2^ While these risks are strongly associated with the degree of prematurity, there are accumulating data to show the importance of nutrition in early life. Macronutrient intakes in the first week of life alone are associated with higher neurodevelopmental scores at 18 months of age,^3^ and data strongly support a positive dose–response relationship between the amount of mother’s own milk an infant receives and later neurodevelopment.^4^ Studies have shown higher verbal intelligence quotient (IQ) in children born preterm who received higher macronutrient intakes in the first four postnatal weeks and these advantages correlate with the size of specific neuroanatomical brain regions using magnetic resonance imaging in adolescents.^5^ These data emphasize the critical importance of establishing adequate nutritional intakes in early life.

Preterm infants present many challenges in establishing appropriate nutrition. While parenteral nutrition is considered a standard of care in most very preterm infants (<32 weeks gestation), it may take several days to achieve adequate intakes.^6^ Enteral milk feeds take several days to establish, and while the benefits of mother’s own milk are unequivocal, unfortified breastmilk will not provide sufficient nutrients to meet the needs of most very preterm infants.^7^ It is therefore common for preterm infants to accumulate nutrient deficits during the hospital stay, and many preterm infants are discharged home on a lower weight and length centile compared to their birth centile.^8^

Studies have therefore explored whether increasing macronutrient intakes post-hospital discharge improve outcomes. Breastfeeding after discharge is strongly recommended and a small number of studies have explored macronutrient supplementation in these infants, but these show no consistent evidence of benefit.^9^ Meta-analysis of trials of infants who are formula feeding after discharge show greater weight gain in infants receiving macronutrient enrichment, but no consistent benefits on neurodevelopment in infancy.^10^ Widely used measures of infant development such as the Bayley Scales of Infant Development (BSID) may lack the sensitivity to detect differences due to nutritional interventions in early life^11^ and assessment of cognition in later childhood is therefore important. The aim of our study was to conduct long-term follow-up of a randomized controlled trial (RCT) of macronutrient enrichment in formula-fed preterm infants following hospital discharge. Follow-up in infancy had shown greater growth at 12 weeks post-term and 18 months of age in the infants who received a macronutrient enriched formula on discharge, but no differences in infant developmental outcome using BSID II.^12^ We aimed to determine the effect of differences in the diet on cognitive outcomes in later childhood and to explore any associations between growth in infancy and later childhood cognition.

## Methods

### Study design

We studied children recruited as preterm infants from a single tertiary neonatal unit in Newcastle upon Tyne, UK between 1993 and 1997, and full details of the original trial are provided elsewhere.^12,13^ Briefly, preterm infants with a mean (±SD) gestation of 31 (±2) weeks and birthweight of 1.37 (±0.3) kg without significant neurological abnormalities or ongoing respiratory disease at discharge were recruited. Parents who had chosen to formula feed were approached prior to hospital discharge at a mean age of 36 corrected weeks, and their infants randomized to one of three different diets. The three diet groups were: (A) preterm formula (PTF) from discharge to 6 months corrected age; (B) term formula (TF) from discharge to 6 months; or (C) PTF from discharge to term corrected age, and TF until 6 months corrected age (the so-called “crossover” group). Growth was assessed regularly using standard techniques until 2 years corrected age and we excluded children who were lost to follow-up before 18 months corrected age. Full details on nutrient compositions are available elsewhere,^13^ but in summary, PTF had an energy density of 80 kcal/100 mL and protein 2.2 g/100 mL, and TF contained 67 kcal/100 mL and 1.6 g/100 mL. For this follow-up study, parents were contacted by letter and children were reviewed at ~10 years of age by one of two psychologists (V.G., G.B.) blinded to original group allocation and infant growth outcomes. Research Ethics approval (Newcastle and North Tyneside Research Ethics Committee) and signed parental consent were obtained.

### Measurements

Cognitive ability was assessed using a short-form version of the Wechsler Intelligence Scale for children 3rd edition (WISC-III) that allows the computation of all four-factor index scores. Each index has a mean score of 100 and SD of 15.^14,15^ Full-scale IQ (FSIQ) was used as a measure of overall cognitive ability and four-factor index scores were determined that relate to specific elements of cognitive functioning. The verbal comprehension index (VCI) evaluates verbal reasoning ability, freedom from distractibility index (FDI) assesses working memory and attention, Perceptual Organization (POI) measures visuospatial reasoning and Processing Speed Index (PSI) tests the speed and accuracy of information processing. An FSIQ score of 80 or above is considered within the normal range, with scores of 70-80 and 55-69 indicating ‘borderline’ and mild cognitive impairment, respectively.

Weight, height and occipitofrontal head circumference (OFC) was measured using standard techniques and standard deviation scores (SDS) calculated using the British 1990 growth references.^16^ To ascertain growth during a specific epoch, weight, length, or OFC change in SDS were calculated by subtracting the SDS at the start of the period from that at the end. We used maternal higher education (i.e., any formal education after school) as a crude proxy for parental education, need for mechanical ventilation in infancy as a proxy of illness severity, and determined socioeconomic status (Townsend index) using a nationally validated deprivation score based on residential locality.^17^

### Statistical analysis

Baseline characteristics were compared using *t* test (parametric) or Mann–Whitney U (nonparametric) tests. Individual linear regression models were generated using FSIQ and the four-factor index scores as separate dependent outcomes to assess associations between diet group or epoch of infant growth and subsequent cognitive outcome. Multivariate regression models were used to quantify associations after adjustment for potential confounding factors. Variables included in the models were sex, gestation, birthweight SDS, Townsend index, and maternal education level. Levels of significance were set at *p* ≤ 0.05, and statistical analysis was performed using STATA version 11.0.

## Results

There were 113 potentially eligible children who had completed assessments at both 18 and 24 months corrected age. We were able to trace, contact, and enroll 92 (81%) of these at a median age of 10.1 years (Table 1). The children who were followed up appeared broadly representative of the original study population with no significant differences in birthweight (*p* = 0.56) or gestational age (*p* = 0.67). The three randomized groups did not differ significantly in demographic or clinical characteristics (Table 1). There were no children with a diagnosis of cerebral palsy or other major neuro-disability. Eight children (9%) were classified as having mild cognitive impairment with an FSIQ score of <70 (two PTF, four TF, and two crossover), and a further eight children had borderline impairment with an FSIQ score of 70–80 (two PTF, two TF, and four crossover). Growth outcomes for the entire cohort split by the study group are provided in Supplementary Table 1.

**Table 1:** Baseline characteristics of the follow-up cohort.

	Total (*n* = 92)	Preterm formula (*n* = 37)	Term formula (*n* = 37)	Crossover group (*n* = 18)
Median age follow-up (years)	10.1 (8.8, 10.6)	10.0 (8.7, 10.6)	10.0 (8.8, 10.5)	10.5 (10.0, 11.5)
Mean birthweight (g)	1373 (0.31)	1422 (0.32)	1342 (0.30)	1333 (0.31)
Mean birthweight SDS	−0.77 (−1.4, −0.18)	−0.73 (−1.38, −0.13)	−0.71 (−1.28, −0.2)	−0.93 (−1.8, −0.17)
Mean gestation (weeks)	30.9 (2.2)	31.0 (2.3)	30.6 (2.2)	31.0 (1.9)
Mean discharge age (weeks)	36.4 (1.48)	36.4 (1.42)	36.3 (1.61)	36.3 (1.37)
Higher maternal education (%)	5.6	2.8	8.3	5.6
Requiring ventilation (%)	55	56	61	44
Median discharge OFC SDS	−0.19 (−0.58, 0.56)	0.17 (−0.43, 0.69)	−0.31 (−0.63, 0.48)	−0.33 (−1.29,0.35)

*OFC* occipitofrontal circumference, *SDS* standard deviation score.

There were no significant differences in cognitive outcomes between PTF and TF groups in either the unadjusted or adjusted results, and no effect of sex or gestational age. Unadjusted mean scores in the crossover group were lower than the PTF and TF groups for FSIQ and for all subsets of the cognitive outcome, but these differences were only significant for the Processing Speed subset, which was 10 points lower in the crossover group compared to the PTF group (*p* = 0.04) (Table 2). Although there were no significant differences between sex, birthweight SDS, gestation, socioeconomic status, or maternal education between groups, the regression analysis was repeated controlling for these factors, as all of these may influence the cognitive outcome. Controlling for these potential confounding factors did not materially change the results, although the lower scores in the crossover group became nonsignificant (Table 3).

**Table 2:** Cognitive outcome full scale and subset scores by diet.

Subtest	Overall mean (SD)	PTF (SD)	TF (SD)	*P* value TF vs. PTF	Crossover (SD)	*P* value crossover vs. PTF
FSIQ	92.7 (14.9)	94.2 (15.6)	93.2 (13.4)	0.70	88.4 (16.5)	0.18
VCI	96.6 (15.3)	99.0 (16.8)	96.6 (14.1)	0.47	91.6 (14.0)	0.12
FDI	95.8 (16.3)	93.0 (20.4)	98.4 (11.7)	0.19	96.3 (14.8)	0.65
POI	91.4 (14.3)	91.4 (13.9)	92.7 (13.3)	0.84	88.5 (17.1)	0.42
PSI	96.0 (15.9)	100.0 (18.0)	95.0 (13.9)	0.12	89.9 (13.3)	0.04

*FDI* freedom from distractibility index, *FSIQ* full-scale intelligence quotient, *POI* perceptual organization index, *PSI* processing speed index, *PTF* preterm formula, *SD* standard deviation, *TF* term formula, *VCI* verbal comprehension index.

**Table 3:** Multivariate associations of cognitive outcome with term formula and crossover groups compared to the preterm formula group.

WISC-III	Term formula			Crossover		
	Coeff	95% CI	*P* value	Co-eff	95% CI	*P* value
FSIQ	−1.05	−8.57, 6.46	0.78	−4.60	−13.68, 4.48	0.32
VCI	-2.41	−10.11, 5.29	0.54	−7.38	−16.69, 1.93	0.12
FDI	4.41	−3.64, 12.46	0.28	3.38	−6.34, 13.11	0.69
POI	1.94	−5.18, 9.05	0.59	−1.57	−10.18, 7.03	0.72
PSI	−6.17	−13.44, 1.11	0.10	−7.74	−16.53, 1.05	0.09

Model adjusted for child’s sex, birthweight SDS, gestation, Townsend index, and maternal education.

*FDI* freedom from distractibility index, *FSIQ* full-scale intelligence quotient, *POI* perceptual organization index, *PSI* processing speed index, *VCI* verbal comprehension index.

After controlling for potential confounders, more rapid weight gain between term and 12 weeks corrected age (“12 weeks”) was significantly associated with a 5-point higher FSIQ (*p* = 0.02), and 4-point higher POI for every unit increase in weight SDS between term and 12 weeks (*p* = 0.04) (Table 4). Weight gain after 12 weeks, that is, for the remainder of the first year of life was not significantly associated with changes in later cognition. There was no significant association between an increase in OFC SDS until 12 weeks age and later cognition, but FSIQ increased by 3.8 points (*p* = 0.04) and the freedom from distractibility index (FDI) increased by six points (*p* = 0.003) for every unit increase in OFC SDS. This impact of OFC growth over the first year of life remained significant for the FDI even after adjusting for weight and length gain (Table 4); however, there were no independent associations between change in length SDS and cognitive outcome.

**Table 4:** Multivariate associations of growth with cognitive ability.

	Adjusted co-eff	95% CI	*P* value
Change in weight SDS (term to 12 weeks)			
FSIQ	5.18	0.75, 9.62	0.02
VCI	3.31	−1.38, 8.00	0.16
FDI	2.10	−2.76, 6.96	0.39
POI	4.36	0.13, 8.58	0.04
PSI	3.46	−1.03, 7.96	0.13
Change in OFC SDS (term to 12 weeks)			
FSIQ	4.27	−1.73, 10.26	0.16
VCI	5.03	−1.12, 11.18	0.11
FDI	4.71	−1.86, 11.27	0.16
POI	2.46	−2.96, 7.88	0.37
PSI	-0.27	−6.15, 5.62	0.93
Change in OFC SDS (term to 1-year CGA)			
FSIQ	3.80	0.06, 7.53	0.04
VCI	2.94	−0.99, 6.86	0.14
FDI	6.17	2.22, 10.13	0.003
POI	1.75	−1.81, 5.30	0.33
PSI	1.86	−1.85, 5.56	0.32
Change in OFC SDS (term to 1-year CGA)^a^			
FSIQ	3.20	−1.56, 7.96	0.19
VCI	3.05	−1.92, 8.02	0.23
FDI	5.23	0.30, 10.17	0.04
POI	1.84	−2.66, 6.33	0.42
PSI	0.96	−3.33, 5.46	0.67

Model adjusted for child’s sex, birthweight SDS, gestation, Townsend index, and maternal education.

*CGA* corrected for gestational age, *CI* confidence interval, *FDI* freedom from distractibility index, *FSIQ* full-scale intelligence quotient, *OFC* occipitofrontal circumference, *POI* perceptual organization index, *PSI* processing speed index, *SDS* standard deviation score, *VCI* verbal comprehension index.

^a^Analysis further adjusted for gain in weight and length over the same period.

## Discussion

We assessed cognitive outcome in children born preterm who were formula fed and recruited to a randomized trial of post-discharge diet. Overall, their mean FSIQ was ~7 points below standardized mean scores, similar to other follow-up studies in preterm infants^,^^18,19^ and we did not detect an impact of the early randomized dietary group. Importantly, we have shown a strong association of early infant growth and later cognition, which was significant for weight gain in the first 12 weeks. Our data also show that head growth in the first year was significantly associated with both FSIQ and the FDI subset, which remained significant even after adjusting for weight gain. These data are in keeping with other studies in preterm infants demonstrating that weight gain between term and 4 months corrected age was associated with higher developmental scores at 18 months^20^ and another study showing that each unit increase in weight SDS between term and 1 year of age was associated with a 1.9-point IQ advantage at 8 years of age.^21^ In combination, these growth data emphasize that the first year of life remains a potentially important period for optimizing brain development through dietary management.^22^ Although there is no evidence of a long-term cognitive benefit for enriched formula after hospital discharge, it is important to note that our children were generally healthy preterm infants, and it is possible that there may be an advantage for higher-risk preterm populations. Some smaller studies show a benefit for macronutrient enrichment in high-risk infants after discharge,^23^ and this might be especially important where infants are unable to upregulate feed volumes to meet caloric demands.

Strengths of our study include the randomized controlled design, detailed measures of early growth, and 10-year follow-up of cognition by trained psychologists blinded to original group allocation and early growth measures. However, our study has important limitations. It was not powered to detect long-term differences in cognition, and while we were able to follow-up >80% of the original cohort 10 years later, it is possible that those followed up are not representative of the entire cohort or other very preterm infants. Furthermore, long-term follow-up studies reflect nutritional practices many years ago, which may be less relevant for contemporary populations. Our study included relatively healthy preterm infants who were not breastfed, yet there is increasing data to show a persisting benefit of breastfeeding after discharge in preterm infants. The associations between infant growth and later cognition are important, but they are observational in nature and may be biased by residual confounding or due to reverse causation, for example, by genetic factors. Our small group sizes precluded analysis by sex, even though the addition of sex into the model did not change the results.

In summary, preterm infants are frequently discharged from the neonatal intensive care unit on a weight centile lower than that at birth, but feeding formula enriched with macronutrients compared to standard milk formula does not appear to improve long-term cognitive outcome in otherwise healthy infants. The strong associations between infant growth and later cognition are important and suggest that nutritional exposures and growth in the first year of life require closer attention.

## Supplementary information

Supplementary Table 1
