# Supplementary material for: Early diet in preterm infants and later cognition: 10-year follow-up of a randomized controlled trial
Source: Pediatr Res. 2021 Feb 9;89(6):1442–6. doi: 10.1038/s41390-021-01368-y (PMC8163596; doi:10.1038/s41390-021-01368-y)
Supplement: Supplementary file 1 — Supplementary Table 1 [file 41390_2021_1368_MOESM1_ESM.docx]

**Supplementary Table 1:Weight, head circumference and growth z-scores by dietary group**

|  | Total (n=92) | Preterm formula (n=37) | Term formula (n=37) | Crossover (n=18) |
| --- | --- | --- | --- | --- |
|  | Mean (standard deviation) | | | |
| **Absolute weight SDS** |  |  |  |  |
| Birth | -0.91(0.93) | -0.82 (0.84) | -0.90 (0.94) | -1.14 (1.12) |
| Discharge | -1.37 (0.95) | -1.15 (0.88) | -1.38 (1.00) | -1.78 (0.88) |
| Term | -0.94 (0.97) | -0.57 (0.87) | -1.13 (0.93) | -1.30 (1.06) |
| T+12 weeks | -0.81 (1.05) | -0.38 (0.95) | -0.92 (0.96) | -1.47 (1.04) |
| 1 year CGA | -0.77 (1.04) | -0.59 (1.12) | -0.76 (0.79) | -1.17 (1.23) |
| **Absolute OFC SDS** |  |  |  |  |
| Discharge | -0.04 (1.06) | +0.21 (0.93) | -0.12 (1.13) | -0.39 (1.11) |
| Term | +0.57 (1.00) | +0.88 (0.95) | +0.37 (0.89) | +0.31 (1.20) |
| T+12 weeks | +0.22 (1.08) | +0.55 (1.03) | +0.09 (1.04) | -0.22 (1.10) |
| 1 year CGA | -0.25 (1.29) | -0.06 (1.19) | -0.36 (1.58) | -0.42 (1.38) |
| **Weight SDS change** |  |  |  |  |
| Disch-term | +0.47 (0.69) | +0.61 (0.59) | +0.26 (0.82) | +0.64 (0.46) |
| Term-12 weeks | +0.12 (0.74) | +0.15 (0.81) | +0.25 (0.70) | -0.21 (0.62) |
| 12 wks- 1yr | +0.05 (0.83) | -0.21 (0.69) | +0.16 (0.90) | +0.35 (0.86) |
| **OFC SDS change** |  |  |  |  |
| Disch-term | +0.57 (0.54) | +0.67 (0.49) | +0.39 (0.64) | +0.75 (0.29) |
| Term-12 weeks | -0.31 (0.60) | -0.32 (0.63) | -0.18 (0.51) | -0.55 (0.64) |
| 12 wks- 1yr | -0.45 (0.71) | -0.61 (0.60) | -0.41 (0.83) | -0.20 (0.60) |

OFC: occipitofrontal circumference, SDS: standard deviation score
